# Using genetics to decipher the link between type 2 diabetes and cancer: shared aetiology or downstream consequence?

**DOI:** 10.1007/s00125-020-05228-y

**Published:** 2020-07-23

**Authors:** Emma E. Vincent, Hanieh Yaghootkar

**Affiliations:** 1 MRC Integrative Epidemiology Unit at the University of Bristol, Bristol, Bristol, UK; 2 Population Health Sciences, Bristol Medical School, University of Bristol, Bristol, UK; 3 School of Cellular and Molecular Medicine, Biomedical Science Building, University of Bristol, Bristol, BS8 1TW UK; 4 Genetics of Complex Traits, University of Exeter Medical School, Royal Devon & Exeter Hospital, Exeter, UK; 5 School of Life Sciences, College of Liberal Arts and Science, University of Westminster, London, UK; 6 Division of Medical Sciences, Department of Health Sciences, Luleå University of Technology, Luleå, Sweden

**Keywords:** Adiposity, Cancer, Diabetes, Genetics, GWAS, Mendelian randomisation, Review, Type 2 diabetes

## Abstract

Recent developments in the field of genetics have accelerated our understanding of the aetiology of complex diseases. Type 2 diabetes mellitus and cancer are no exception, with large-scale genome-wide association studies (GWAS) facilitating exploration of the underlying pathology. Here, we discuss how genetics studies can be used to investigate the relationship between these complex diseases. Observational epidemiological studies consistently report that people with type 2 diabetes have a higher risk of several types of cancer. Indeed, type 2 diabetes and cancer share many common risk factors, such as obesity, ageing, poor diet and low levels of physical activity. However, questions remain regarding the biological mechanisms that link these two diseases. Large-scale GWAS of type 2 diabetes and cancer allow us to consider the evidence for shared genetic architecture. Several shared susceptibility genes have been identified, yet tissue specificity and direction of effect must be taken into account when considering common genetic aetiology. We also consider how GWAS, and associated techniques such as Mendelian randomisation, allow us to dissect the link between the two diseases and address questions such as ‘Does type 2 diabetes cause cancer or is the increased risk observed driven by higher adiposity or another associated metabolic feature?’

**Electronic supplementary material:**

The online version of this article (10.1007/s00125-020-05228-y) contains a slideset of the figures for download, which is available to authorised users.

**Figure:**
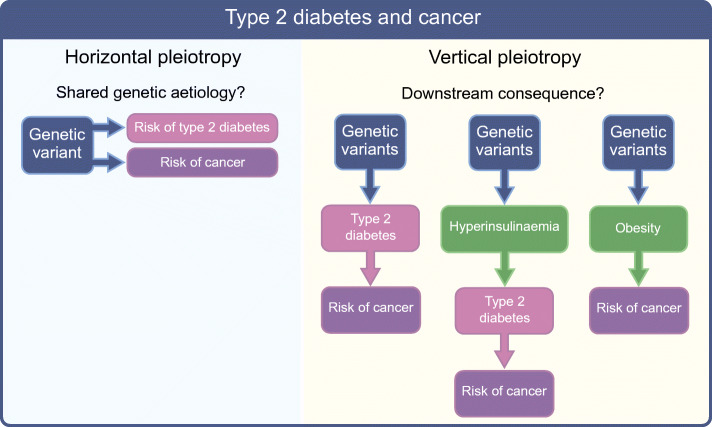
Graphical abstract

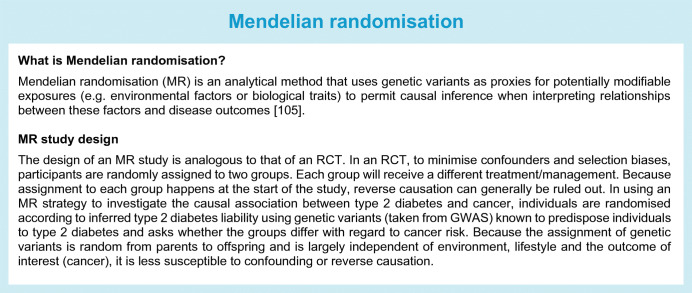

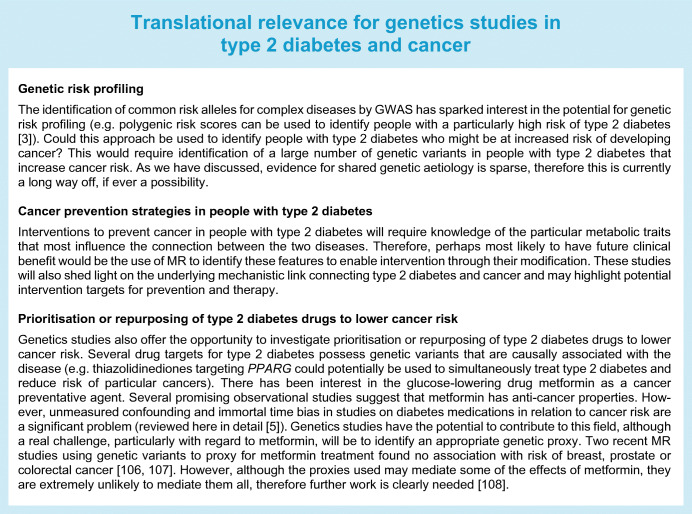





## Introduction

Of the 4.7 million people in the UK (>450 million worldwide) with diabetes, ~90% have type 2 diabetes mellitus [1]. Observational epidemiological studies have consistently reported that people with type 2 diabetes have a higher risk of certain types of cancer (not prostate cancer, where an inverse relationship has been reported) [2]. Although type 2 diabetes is typically diagnosed by elevated levels of circulating glucose, it presents as a collection of metabolic features, some of which may influence cancer development whereas others may not. To date, the biological mechanisms principally hypothesised to support associations between type 2 diabetes and cancer include hyperglycaemia, hyperinsulinaemia, sex hormone dysregulation and chronic low-grade inflammation [2]. However, questions remain as to the exact features of type 2 diabetes that drive the association and whether they are causal.

**Fig. 1:**
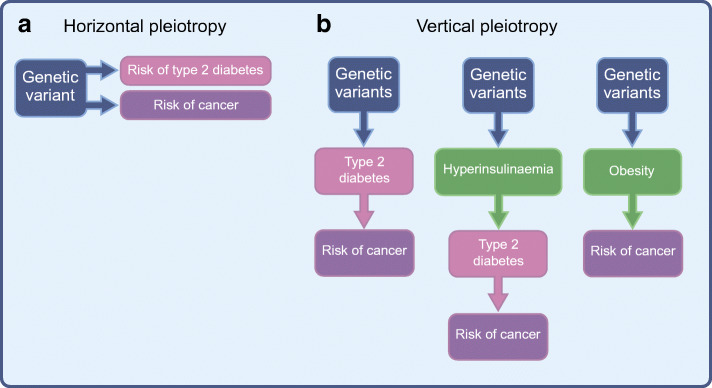
The relationship between type 2 diabetes and cancer. (**a**) The increased risk of cancer in people with type 2 diabetes could be due to shared genetic aetiology between the two diseases. In this scenario, genetic variants that predispose individuals to type 2 diabetes would also predispose individuals to cancer. The relationship between type 2 diabetes and cancer is therefore horizontal, as the effect of the genetic variant on each disease is independent and is exerted through different mechanisms. (**b**) The increased risk of cancer in people with type 2 diabetes may be due to a tumorigenic effect driven by either type 2 diabetes itself or an associated trait (such as adiposity). This is an example of vertical pleiotropy, as there is a linear pathway from type 2 diabetes (or associated trait) to cancer. MR uses genetic variants as a proxy for the exposure of interest to assess whether there is a vertical causal pathway from the exposure to the outcome. This figure is available as part of a downloadable slideset

Here, we focus on the contribution to this field made by genetics studies and on the insights these studies have provided in elucidating the link between type 2 diabetes and certain types of cancer. First, we discuss whether shared genetic aetiology may explain the increased risk of cancer in people with type 2 diabetes (i.e. horizontal pleiotropy: genetic variants associated with type 2 diabetes are also independently [via different mechanisms] associated with cancer risk). Second, we consider the evidence for vertical pleiotropy (i.e. a causal path from type 2 diabetes [or a particular metabolic feature of type 2 diabetes] to cancer development) by appraising the evidence from Mendelian randomisation (MR) studies (Fig. 1 and Text box: Mendelian randomisation).

## Horizontal pleiotropy: do type 2 diabetes and cancer share common genetic aetiology?

Since the advent of genome-wide association studies (GWAS) in 2005, major progress has been made in our understanding of complex diseases including type 2 diabetes and cancer [3, 4]. Type 2 diabetes itself and the cancer types observationally associated with it are relatively common, yet high-impact variants that cause monogenic forms of diabetes or heritable cancer syndromes are exceedingly rare and tend to result in early-onset disease. GWAS have revealed that the inherited contribution to more prevalent, later-onset disease is comprised of many common variants, with each individual variant having a relatively small impact [3]. Here, we discuss whether common risk alleles predispose to both type 2 diabetes and particular cancers and whether this can explain the observed association between type 2 diabetes and particular cancer types. We discuss examples that provide the most convincing evidence for shared genetic aetiology between the two diseases, discuss shared susceptibility genes and highlight scenarios that may result in misinterpretation of common aetiology.

### Evidence for type 2 diabetes risk alleles increasing risk of cancer

Several types of cancer, including liver, pancreatic, endometrial, breast, colorectal, bladder and kidney cancer and non-Hodgkin’s lymphoma, have been positively and observationally associated with type 2 diabetes [5]. However, evidence for shared genetic aetiology between type 2 diabetes and these cancers is scarce. Several type 2 diabetes susceptibility genes are associated with cancer development, yet this does not necessarily imply shared genetic aetiology. This would be demonstrated by the identification of the same genetic variant and risk allele (or those in linkage disequilibrium) that independently predisposes to both type 2 diabetes and cancer (Fig. 1a). Genetic variation at *TCF7L2* (transcription factor 7 like 2) is perhaps the best and most extensively studied example of this.

The transcription factor encoded by *TCF7L2* operates at the last stage of the canonical Wnt signalling transduction cascade [6]. Genetics studies have reported a positive association between type 2 diabetes predisposing alleles within *TCF7L2* and cancer including colorectal [7–9], breast [10–12] hepatocellular [13] and aggressive prostate cancer [14]. A recent comprehensive meta-analysis found type 2 diabetes risk alleles at eight variants in *TCF7L2* were associated with increased risk of breast, colorectal and lung cancer and glioma [15].

The association between type 2 diabetes risk alleles in *TCF7L2* and higher risk of colorectal cancer has been replicated in several studies [7, 16, 17]. The Wnt pathway is a major driver of colorectal carcinogenesis and the *TCF7L2* gene is frequently mutated in colorectal cancer [18]. The association with colorectal cancer has been shown to be independent of type 2 diabetes or obesity [7, 16, 17], suggesting the risk alleles are likely to have greatest impact specifically in colon tissue and not in the pancreatic islet (which is likely to mediate the impact of the variants on type 2 diabetes).

Studies investigating a number of different type 2 diabetes predisposing variants in relation to cancer risk have reported only a small number of weak associations and findings are often inconsistent. In one study, among 37 type 2 diabetes risk alleles, two (in *FTO* and *MTNR1B*) showed nominally positive associations with pancreatic cancer risk and one showed an inverse association (in *BCL11A*) [19]. In another, among 33 type 2 diabetes risk alleles, three (in *FTO*, *TCF7L2* and *PRC1*) were found to be associated with breast cancer [20]. Inconsistently, two additional studies have shown null associations with breast cancer across all type 2 diabetes variants tested [21, 22].

### Directionality of type 2 diabetes risk alleles on cancer risk

Observational epidemiology shows several cancers to be positively associated with type 2 diabetes; however, prostate cancer has been consistently found to be inversely associated. In agreement, several type 2 diabetes risk alleles have been found to associate with reduced risk of prostate cancer. Type 2 diabetes risk alleles in *PPARG* (encoding peroxisome proliferator-activated receptor γ [PPARγ]) [23, 24] and *HNF1B* (encoding hepatocyte nuclear factor 1 homeobox B) [25] lower the risk of prostate cancer. One study found that 10 out of 36 type 2 diabetes risk alleles were nominally inversely associated with prostate cancer, with only the *HNF1B* risk allele remaining significant following multiple testing correction [26]. Another study found that four of 13 type 2 diabetes predisposing alleles were nominally inversely associated [27]. Using a genetic risk score (GRS) consisting of 18 type 2 diabetes risk variants, it has been shown that people with higher genetic susceptibility to type 2 diabetes have a reduced risk of prostate cancer [28]. However, some studies have found no association between type 2 diabetes risk variants (either individually or in risk scores) and prostate cancer [29, 30].

Genetic variants in *JAZF1* (encoding JAZF zinc finger 1) also have an inverse effect on the relationship between type 2 diabetes and prostate cancer. A recent GWAS found two genetic variants in *JAZF1*, one with a major allele predisposing to type 2 diabetes [31] and the other with a major allele protecting against prostate cancer [32]. As the genetic variants are not correlated (not in linkage disequilibrium), they may operate through different pathways to alter type 2 diabetes and prostate cancer risk, respectively. This example highlights how genetic variation in the same gene can affect the risk of two diseases without necessarily meaning that the diseases share genetic aetiology.

The direction of effect of type 2 diabetes risk alleles on cancer is often not clear-cut, with some type 2 diabetes predisposing alleles in the same gene increasing the risk of one cancer but protecting against another. For example, type 2 diabetes risk alleles in *PPARG* are associated with elevated risk of pancreatic cancer [33] and reduced risk of colorectal cancer [34]. This pattern of association suggests the role of *PPARG* likely depends on the context and should be interpreted accordingly. This finding is also consistent with the described multifaceted role of PPARγ in cancer, wherein it exhibits both tumour-suppressive and tumour-promoting properties [35]. Similar evidence comes from *HNF1B*, for which type 2 diabetes risk alleles are associated with ovarian cancer but the direction of effect varies by subtype (serous vs clear cell) [36, 37].

### Shared susceptibility genes: directionality, tissue specificity and context

Several common susceptibility genes have been implicated in type 2 diabetes and particular cancers but there is currently little evidence for shared genetic aetiology for many of these. For example, common variants in *KLF14* (encoding Kruppel-like factor 14, an imprinted transcription factor), associated with lower expression in adult adipose tissue, cause a defect of adipogenesis that is likely to reflect impaired glucose uptake and consequently higher risk of type 2 diabetes [38]. *KLF14* expression is reduced in many types of human cancer, including breast, lymphatic, cervical, oral cavity, floor of mouth, pancreas and colorectal cancers [39]. Despite reduced expression having been associated with both type 2 diabetes and certain types of cancer, further studies are needed to investigate whether type 2 diabetes predisposing alleles resulting in reduced expression of *KLF14* are also associated with risk of the implicated cancers.

GWAS of type 2 diabetes have consistently identified variants (or colocalising variants) in genes implicated in proliferation and cell cycle regulation [40, 41]. These hallmarks are traditionally associated with cancer; however, direction of effect and tissue specificity are key here. As an illustration, type 2 diabetes risk alleles identified at the *CDKN2A/B* locus [41] have been implicated in reduced beta cell function [42, 43]. Transgenic mice overexpressing *CDKN2A/B* display decreased islet proliferation [44], suggesting that the type 2 diabetes risk alleles influence pathology by increasing *CDKN2A/B* expression. Conversely, rare *CDKN2A* loss-of-function mutations cause familial melanoma and individuals carrying these mutations have improved beta cell function [45]. The direction of effect here is clearly opposing and even though the gene is implicated in both diseases, the context is vastly different and they would not be expected to share risk alleles at these loci. An important consideration when evaluating evidence for shared genetic aetiology is that many type 2 diabetes predisposing variants are in pancreatic islet enhancers and are therefore unlikely to be implicated in cancer development in other tissues.

Furthermore, type 2 diabetes predisposing variants in genes involved in the insulin signalling pathway are expected to have an opposing direction of effect on cancer. Variants in *AKT2* and *PTEN* have been associated with type 2 diabetes risk; their presence reduces activity of the insulin signalling pathway, leading to reduced glucose uptake and insulin resistance in insulin-responsive tissues. Conversely, tumour cells would typically benefit from enhanced activity of this pathway (through pro-proliferative and anti-apoptotic signals). Indeed, *PTEN* (a negative regulator of the pathway) and *AKT2* are well-characterised tumour suppressor and oncogenes, respectively. Indeed, loss-of-function mutations in *PTEN* cause a rare cancer-predisposition syndrome and protect against type 2 diabetes (through enhanced insulin sensitivity) [46].

### Summary

Several type 2 diabetes susceptibility genes are known to play a role in cancer development (e.g. *TCF7L2*, *CDKN2A/B*, *AKT2*, *PPARG*, *PTEN* and *HNF1B*) but the evidence is relatively scarce for shared genetic aetiology between type 2 diabetes predisposing alleles and the observationally associated cancers. However, there are particular contexts wherein the evidence appears consistent and persuasive: the positive association between type 2 diabetes risk alleles in *TCF7L2* and higher risk of breast and colorectal cancer; and the association between type 2 diabetes predisposing alleles and lower risk of prostate cancer.

What quickly becomes apparent when appraising the role of type 2 diabetes susceptibility genes in the development of a particular cancer is that the situation is very rarely clear-cut. Several genes are implicated in both diseases yet in most cases type 2 diabetes predisposing alleles have not been found to be associated with a cancer and often the biological impact of these alleles would be expected to protect against, rather than predispose to, cancer. When evaluating evidence from genetics studies, it is important to consider that in many of the studies the identity of the gene (or genes) through which the type 2 diabetes predisposing variants act has been assumed. The gene is easy to identify if the variant is in a coding region but most loci are in non-protein coding regions of the genome, making it difficult to determine how the variant impacts disease development. While some elegant work is being carried out in this area [47], further work is needed in the functional annotation of type 2 diabetes predisposing variants. Extrapolating this to determine how the gene may behave in the context of cancer is further complicated by the fact that cancer is very much not one disease. Cancers developing at different sites (and indeed different cancer subtypes developing at the same site) will be exposed to different environments, acquire different mutations and will invariably develop differently. Therefore, directionality, tissue specificity and context are key considerations when assessing evidence for shared genetic aetiology between type 2 diabetes and a particular cancer.

## Vertical pleiotropy: using genetic variants to investigate a causal pathway to cancer through type 2 diabetes

Despite the lack of convincing evidence that shared genetic aetiology explains the observed positive association between type 2 diabetes and risk of a particular cancer, an observational correlation between diabetes and cancer quite clearly exists. To explore this further, we will appraise the evidence gained from genetics studies employing an MR strategy for a vertical (causal) pathway from type 2 diabetes (or a particular metabolic feature of type 2 diabetes) to cancer (Fig. 1b).

Determining whether there is a causal relationship between type 2 diabetes and cancer has been challenging. Observational epidemiological studies are limited due to certain biases including confounding, measurement error and reverse causation. Confounders are factors that may independently influence both the risk of type 2 diabetes and risk of cancer (e.g. alcohol consumption, adiposity and lower socioeconomic status). Reverse causation is also an important consideration, since the development of certain cancers can precede and cause the development of type 2 diabetes (e.g. pancreatic and liver cancer [problems with traditional epidemiological studies in this context are discussed further here [5, 48]]).

**Fig. 2:**
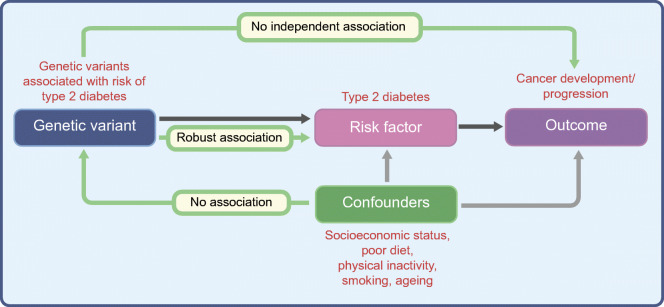
The assumptions of the MR approach. To perform MR, three assumptions (green arrows) need to be satisfied. First, the genetic variants used to proxy the risk factor need to be robustly associated with it. Second, these variants need to be independent of any confounding factors. Third, the variants should only be associated with the outcome through their effect on the risk factor. This figure is available as part of a downloadable slideset

The gold standard study design for inferring causality is a randomised controlled trial (RCT), but it would be ethically unsound and methodologically unfeasible to apply this framework to the question of whether type 2 diabetes causes cancer. A safe, inexpensive alternative is to use a genetic approach: MR [49], essentially a genetic analogue of the RCT [3] (Fig. 2 and Text box: Mendelian randomisation). This powerful use of genetics allows researchers to consider how key environmental and lifestyle factors (e.g. type 2 diabetes) influence complex diseases (e.g. cancer). Certain assumptions must be satisfied in order to perform MR (Fig. 2) and there are particular limitations and considerations when performing MR with cancer as the outcome (comprehensively reviewed elsewhere [50]).

Several studies have used MR to investigate whether there is a causal association between type 2 diabetes and cancer. These studies typically use a GRS composed of type 2 diabetes predisposing genetic variants weighted according to their effect on type 2 diabetes risk. These studies are in agreement in that they find no evidence for an association between the two diseases [51–55], consistent with the lack of evidence from studies assessing the association of single type 2 diabetes predisposing genetic variants and cancer.

Although MR studies do not support a role for type 2 diabetes in cancer development, a number of factors should be considered when interpreting these findings. First, power for an MR study is influenced by the statistical power of the GWAS used. Sample sizes are lower for cancer GWAS than for cardiometabolic disease GWAS, for instance, due to the rarer nature of the disease. Lack of power may limit the ability to detect smaller causal effects using MR. Second, some type 2 diabetes genetic variants have a paradoxical association with the classically defined metabolic features of type 2 diabetes (e.g. a variant in *KCNQ1* is associated with both hyperglycaemia and hypoinsulinaemia [52]). These paradoxical associations could potentially cancel each other out. Finally, and most importantly, type 2 diabetes is a markedly complex and heterogeneous disease. It is characterised by a collection of metabolic events and each affected individual presents with a different contribution of metabolic features. Therefore, the observational association between type 2 diabetes and cancer may be driven by a particular metabolic trait of type 2 diabetes or by an associated condition, such as obesity. Below, we discuss the important metabolic traits thought to influence cancer risk in conjunction with evidence from MR studies.

### Hyperglycaemia

The diagnostic hallmark of type 2 diabetes is hyperglycaemia. Several observational epidemiological studies have reported a positive correlation between fasting glucose levels and risk of and survival from particular cancers [56–60], even in people without diabetes [57, 59]. Tumour cells preferentially use glucose as their main source of energy and become ‘addicted’ to the pathways processing the sugar in the cell. However, there is little evidence to suggest that tumour cells benefit energetically from hyperglycaemia over and above normoglycaemic conditions [61]. Consistent with this, a meta-analysis of RCTs of intensified glycaemic control did not find evidence of any cancer risk reduction in people with type 2 diabetes [62].

Similarly, MR studies have been unable to support a role for hyperglycaemia in cancer development. Genetic variants inferring lifelong differences in fasting glucose were not associated with lung [63], endometrial [53] or pancreatic cancer [51] or renal cell carcinoma [54]. However, genetic variants inferring differences in 2 h (post-challenge) glucose were positively associated with breast cancer, despite there being no association with fasting glucose in the same study [64]. While this suggests that postprandial glucose levels may be pertinent for cancer development, further investigation is required.

### Hyperinsulinaemia

Several observational epidemiological studies have demonstrated that high levels of endogenous insulin are associated with higher risk of cancer incidence and mortality, including risk of colorectal [65], endometrial [66, 67], prostate [68] and breast cancer [69, 70] and breast cancer mortality [71]. Unlike the findings with hyperglycaemia, MR studies support a causal association between higher levels of fasting insulin and risk of endometrial [53], breast [64], lung [63] and pancreatic cancer [51] and renal cell carcinoma [54]. With the exception of higher body fatness, this is by far the most consistent association between a type 2 diabetes-related trait and particular cancers in studies using an MR framework, although replication studies confirming the association found for particular cancers are lacking.

There are several explanations for the causal role of insulin, directly or indirectly, in cancer incidence and risk of mortality. Insulin and insulin-like growth factor 1 (IGF-1) may locally contribute to tumour cell proliferation [72]. Observational epidemiological studies have reported a positive link between circulating IGF-1 levels and increased risk of particular cancers, although associations vary between sites [73]. Evidence from MR studies implicate circulating IGFs in breast cancer [74] and prostate cancer risk and progression [75, 76]. Another purported mechanism is that hyperinsulinaemia inhibits the production of sex hormone binding globulin (SHBG) in the liver, resulting in an elevation of free hormones (including oestrogen and testosterone), which are pro-proliferative and anti-apoptotic [77]. Consistent with this, MR studies have reported an association between sex hormones and puberty timing and breast and endometrial cancer in women [78–80] and between puberty timing and prostate cancer in men [78].

### Adiposity

Among the risk factors shared by type 2 diabetes and cancer, the most dominant and the most likely to confound observational epidemiology is adiposity. Evidence from observational studies suggests there is a positive relationship between excess body fatness and the risk of 13 cancer types [81]. In support of this, observational studies of weight loss [82] and follow-up studies of patients undergoing bariatric surgery [83] show that weight reduction lowers cancer risk.

Several MR studies support a causal association between higher body fatness and risk of six obesity-related cancers [84], renal cell carcinoma [54], endometrial [85], ovarian [55, 86, 87], oesophageal [88], pancreatic [51, 89, 90] and colorectal cancer [87, 91] and reduced survival in oestrogen receptor-positive (ER+) breast cancer [92]. Higher body fatness has also been found to be causally associated with lung cancer [63, 87], a cancer not observationally associated with higher adiposity or type 2 diabetes. The same study also found a positive association between fasting insulin levels and lung cancer, which may mean we need to re-evaluate the cancers we think of as being associated with metabolic dysregulation.

The weight of evidence therefore suggests there is a causal path between higher adiposity and risk of particular cancers. Effort will now need to be directed into investigating which aspects of excess adiposity are important. Adiposity is a complex phenotype and is associated with considerable metabolic and endocrine abnormalities, therefore it may be metabolic dysfunction that increases the risk of cancer, rather than a higher level of body fat per se. In support of this, despite several metabolic features of type 2 diabetes being found to be positively associated with breast cancer, higher adiposity has been found to have a protective effect in MR studies of both pre- and postmenopausal women [64, 87, 93, 94]. This suggests that the association between metabolic features such as increased fasting insulin levels and breast cancer risk may be independent of higher adiposity. However, observational studies have consistently reported a positive association between high BMI and postmenopausal breast cancer, further emphasising that the relationship between BMI and breast cancer is not straightforward. How weight is gained and when across the life course may be important; there is strong evidence from a recent MR study of a protective effect for larger body size in early life with breast cancer risk, with little evidence for an association with adult body size [95].

MR provides a means to uncouple the impact of higher adiposity from its often associated metabolic dysfunction by using genetic variants associated with ‘favourable adiposity’. The adiposity-increasing alleles at these loci have a paradoxical effect on adiposity and risk of cardiometabolic disease [96–98] and are associated with a favourable metabolic profile. Studies using MRI scans of abdominal fat suggest that the underlying mechanism involves an ability to store excess fat in a safe place (subcutaneous adipose tissue), which protects against fat accumulation in ectopic organs like the liver [98]. Employing MR studies to consider the role of favourable vs unfavourable adiposity in cancer risk will indeed be intriguing.

## Conclusions and future work

Here, we have considered the evidence from genetics studies that supports the observed association between type 2 diabetes and particular cancers. It is important to consider these studies in the context of the broader scientific literature. Thorough investigation of the link between type 2 diabetes and particular cancers requires the accumulation of several strands of evidence from studies across diverse methodologies including triangulating evidence across observational and genetic epidemiological studies and animal and laboratory-based studies. Indeed, observational and genetic epidemiological studies have different biases, strengths and weaknesses. Hence, if results are in agreement across different methodologies, they are more likely to be robust [99].

**Fig. 3:**
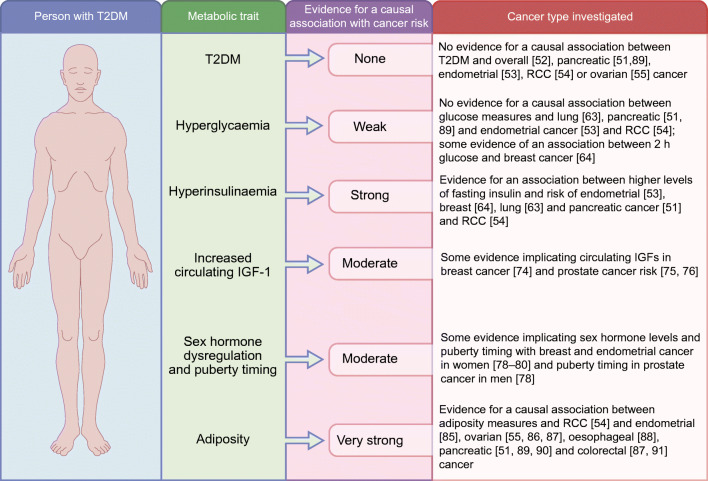
Summary of evidence from MR studies supporting an association between type 2 diabetes (or associated metabolic traits) and cancer. Type 2 diabetes is a complex heterogeneous disease, characterised by a collection of metabolic events and each affected individual presents with a different contribution of metabolic features. MR allows us to consider the causal relationship between type 2 diabetes (when considered as a whole and also each metabolic trait in isolation) and cancer. This approach allows us to investigate whether a particular metabolic trait is responsible for the increased risk of cancer in people with type 2 diabetes. In the figure, the strength of evidence supporting a causal association is defined as follows: none, no evidence for a causal association between trait and cancer; weak, only one study reports a causal association between trait and cancer; moderate, more than one study reports a consistent causal association; strong, several studies report a consistent causal association but replication is limited; very strong, several studies report a consistent causal association and several studies have replicated analyses in the same and some in different cohort data sets. RCC, renal cell carcinoma; T2DM, type 2 diabetes mellitus. This figure is available as part of a downloadable slideset

Based on appraisal of the available evidence from genetics studies it seems unlikely that the observational association between type 2 diabetes and cancer risk is driven by shared genetic aetiology. It seems more likely to be driven by a particular metabolic feature of type 2 diabetes itself, such as increased fasting insulin levels, or by an associated trait, such as higher adiposity, as demonstrated by MR studies (summarised in Fig. 3). However, further work is needed to fully define this complex relationship and to elucidate the underlying biological mechanisms. Recently, interest has grown in deconstructing or partitioning the type 2 diabetes GRS, grouping the genetic variants in relation to their biological role [100–102]. MR can then be employed to assess the causal association between the groups and various cancer types. This could help shed further light on what aspects of this heterogeneous disease are most influential in cancer development and help unpick the underlying mechanisms. In future studies it might also be possible to use MR to explore the potential of particular therapeutic strategies (see Text box: Translational relevance for genetics studies in type 2 diabetes and cancer). It is also likely that epigenetic alterations (driven by type 2 diabetes or associated metabolic features) play a role in promoting cancer development and genetics studies have the potential to help us explore this further [103].

There are several areas that need further exploration. In particular, genetics studies of type 2 diabetes and cancer progression are largely lacking. A recent analysis where this has been attempted used 143 type 2 diabetes predisposing variants and investigated their association with risk of mortality in ~800 people. Twelve SNPs were associated with risk of breast cancer, three with all-cause mortality and three with breast cancer specific mortality, yet none of the associations were significant after multiple testing corrections [104]. Until data from larger cohorts are available, power is likely to be an issue in such studies. Although a handful of MR studies have been undertaken on type 2 diabetes (or related traits) and cancer progression, methodological challenges remain, such as selection bias (discussed further here [50]).

## Electronic supplementary material

Slideset of figures (PPTX 431 kb)

## Abbreviations

GRS: Genetic risk score
GWAS: Genome-wide association studies
HNF1B: Hepatocyte nuclear factor 1 homeobox B
IGF-1: Insulin-like growth factor 1
JAZF1: JAZF zinc finger 1
KLF14: Kruppel-like factor 14
MR: Mendelian randomisation
PPARγ: Peroxisome proliferator-activated receptor γ
RCT: Randomised controlled trial
TCF7L2: Transcription factor 7 like 2
