# Supplementary material for: Using genetics to decipher the link between type 2 diabetes and cancer: shared aetiology or downstream consequence?
Source: Diabetologia. 2020 Jul 23;63(9):1706–17. doi: 10.1007/s00125-020-05228-y (PMC7406536; doi:10.1007/s00125-020-05228-y)
Supplement: Supplementary file 1 — (PPTX 431 kb) [file 125_2020_5228_MOESM1_ESM.pptx]

## Slide 1
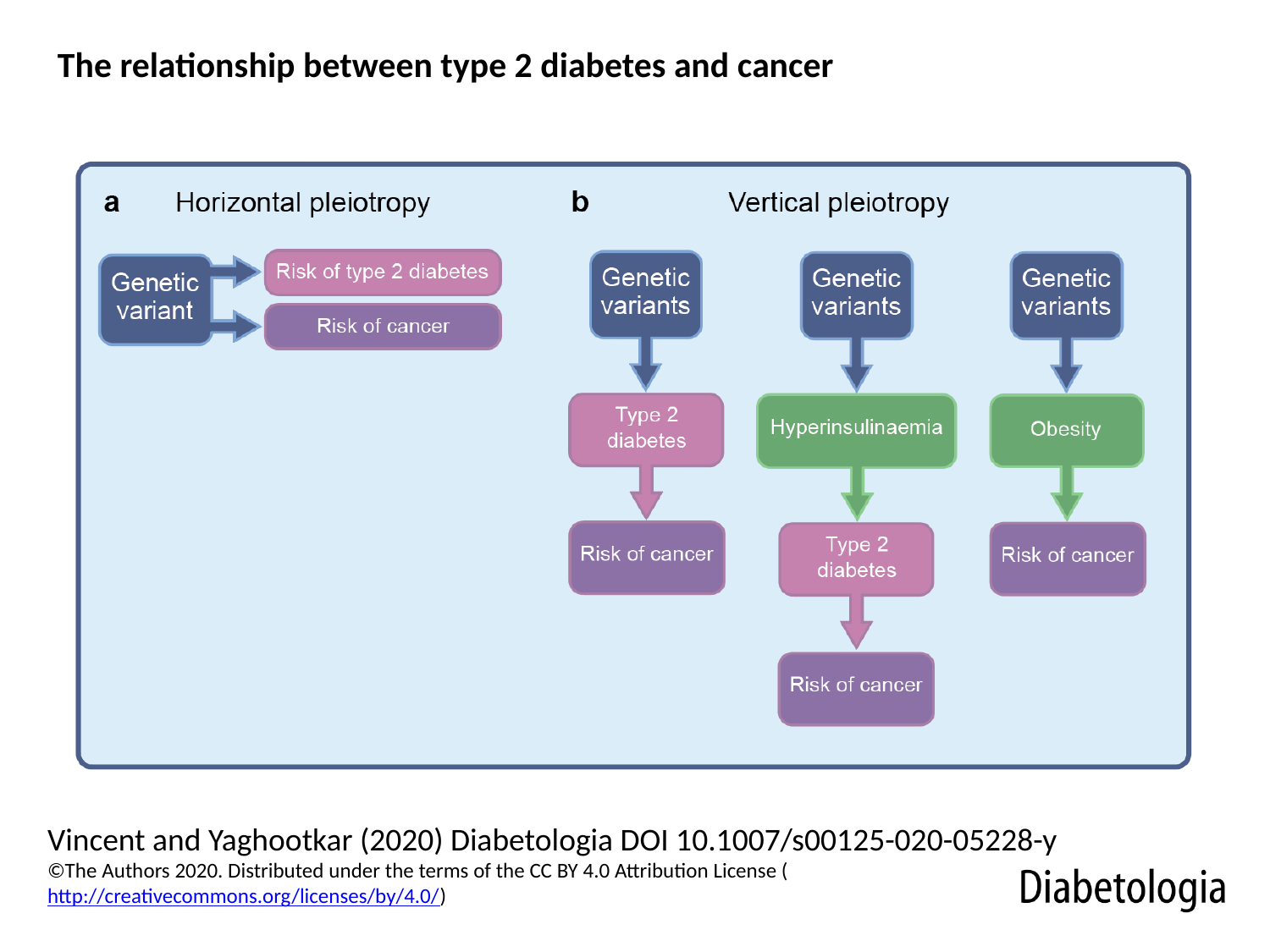

The relationship between type 2 diabetes and cancer
Vincent and Yaghootkar (2020) Diabetologia DOI 10.1007/s00125-020-05228-y
©The Authors 2020. Distributed under the terms of the CC BY 4.0 Attribution License (http://creativecommons.org/licenses/by/4.0/)

## Slide 2
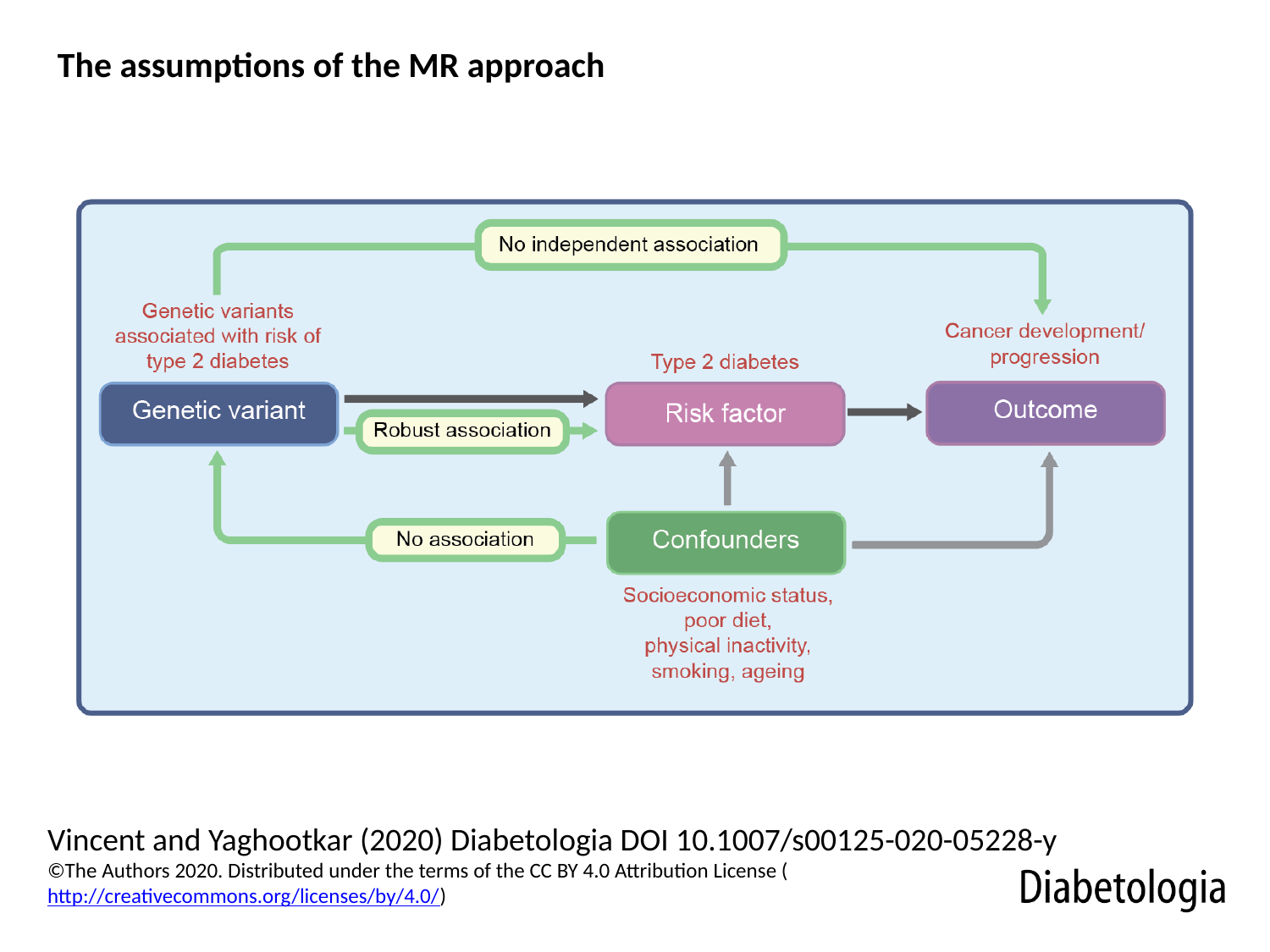

The assumptions of the MR approach
Vincent and Yaghootkar (2020) Diabetologia DOI 10.1007/s00125-020-05228-y
©The Authors 2020. Distributed under the terms of the CC BY 4.0 Attribution License (http://creativecommons.org/licenses/by/4.0/)

## Slide 3
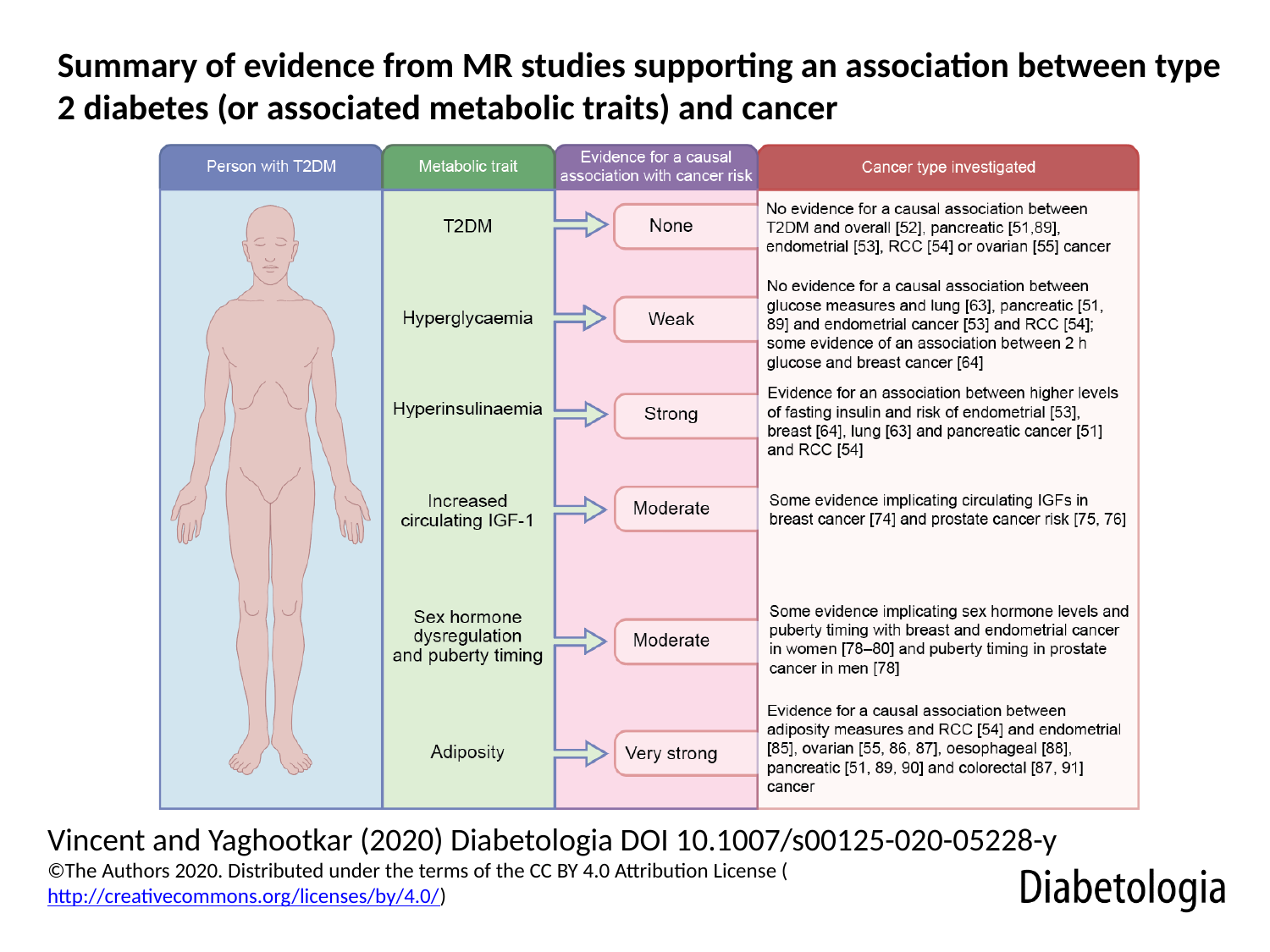

Summary of evidence from MR studies supporting an association between type 2 diabetes (or associated metabolic traits) and cancer
Vincent and Yaghootkar (2020) Diabetologia DOI 10.1007/s00125-020-05228-y
©The Authors 2020. Distributed under the terms of the CC BY 4.0 Attribution License (http://creativecommons.org/licenses/by/4.0/)
